# Src-family kinases negatively regulate NFAT signaling in resting human T cells

**DOI:** 10.1371/journal.pone.0187123

**Published:** 2017-10-26

**Authors:** Alan Baer, Winston Colon-Moran, Jinhua Xiang, Jack T. Stapleton, Nirjal Bhattarai

**Affiliations:** 1 Division of Cellular and Gene Therapies, Office of Tissues and Advanced Therapies, Center for Biologics Evaluation and Research, Food and Drug Administration, Silver Spring, Maryland; 2 Research Service, Iowa City Veterans Affairs Medical Center, and the Department of Internal Medicine, University of Iowa, Iowa City, IA; Hungarian Academy of Sciences, HUNGARY

## Abstract

T cell signaling is required for activation of both natural and therapeutic T cells including chimeric antigen receptor (CAR) T cells. Identification of novel factors and pathways regulating T cell signaling may aid in development of effective T cell therapies. In resting human T cells, the majority of Src-family of tyrosine kinases (SFKs) are inactive due to phosphorylation of a conserved carboxy-terminal tyrosine residue. Recently, a pool of enzymatically active SFKs has been identified in resting T cells; however, the significance of these is incompletely understood. Here, we characterized the role of active SFKs in resting human T cells. Pharmacologic inhibition of active SFKs enhanced distal TCR signaling as measured by IL-2 release and CD25 surface expression following TCR-independent activation. Mechanistically, inhibition of the active pool of SFKs induced nuclear translocation of NFAT1, and enhanced NFAT1-dependent signaling in resting T cells. The negative regulation of NFAT1 signaling was in part mediated by the Src-kinase Lck as human T cells lacking Lck had increased levels of nuclear NFAT1 and demonstrated enhanced NFAT1-dependent gene expression. Inhibition of active SFKs in resting primary human T cells also increased nuclear NFAT1 and enhanced NFAT1-dependent signaling. Finally, the calcineurin inhibitor FK506 and Cyclosporin A reversed the effect of SFKs inhibition on NFAT1. Together, these data identified a novel role of SFKs in preventing aberrant NFAT1 activation in resting T cells, and suggest that maintaining this pool of active SFKs in therapeutic T cells may increase the efficacy of T cell therapies.

## Introduction

T cell receptor (TCR) activation is the first step in generating an effective T cell response [1–3]. Engagement of the TCR with an antigenic peptide bound to the MHC complex present on the surface of antigen-presenting cells (APCs) initiates a series of intracellular signaling events culminating in expression of pleotropic cytokines (IL-2, IFN-γ etc.), and signal transducing receptors (IL-2 receptor alpha; CD25) [1–4]. Persistent signaling through the TCR is detrimental, leading to T cell exhaustion and impaired T cell function [5, 6]. Thus, cells have numerous mechanisms to regulate TCR signaling and maintain T cell homeostasis [7–13].

The activation of two major Src-family tyrosine kinase (SFKs) member (Lck and Fyn) are required for signaling through the TCR [1, 2, 13–15]. In resting T cells, Lck and Fyn are phosphorylated at the carboxy-terminal tyrosine residue (Y505 for Lck and Y528 for Fyn) by the C-terminal Src kinase (Csk) [2, 13, 16]. SFKs phosphorylated at the carboxy-terminal tyrosine maintain a closed conformation that is enzymatically inactive [13, 17, 18]. Upon TCR engagement SFKs are dephosphorylated resulting in a conformational change that allows *trans* autophosphorylation of the tyrosine residue in the kinase domain (Y394 for Lck and Y417 for Fyn) [2, 13, 17, 18]. CD45 is a major phosphatase involved in the dephosphorylation of SFKs; however, other phosphatases may also play a role. SFKs phosphorylated at Y394 or Y417 maintain an open conformation, are enzymatically active and mediate downstream TCR signaling [1–3, 13, 14, 19].

The role of SFKs (Lck/ Fyn) in initiating membrane proximal TCR signaling is well defined and extensively studied [1, 13, 20–22]. Recent studies identified a pool of active Lck and Fyn in resting T cells [2, 14, 23–25], and suggest that this pool contributes to proximal TCR signaling [14]. In addition, active Fyn kinase phosphorylates the Csk-binding protein (Cbp) in resting T cells, which is required for Csk interactions with the Cbp [26]. Csk bound to the phosphorylated Cbp mediates phosphorylation of the carboxy-terminal tyrosine residue of SFKs and inhibits their kinase activity in resting T cells [26]. However, Cbp-deficient mice did not show any developmental defect and the T cell response in these mice were normal [27, 28], suggesting either that Cbp is dispensable, or that other cellular factors compensate for loss of Cbp in T cells for T cell activation. Previous studies found that pharmacologic inhibition of SFKs or genetic knockdown of Lck in T cell lines results in augmented distal TCR signaling [29, 30]. Although, these studies suggest that active SFKs may play a role in distal TCR signaling, the mechanism and significance of SFK-mediated regulation of distal TCR signaling remains unclear.

Nuclear factor of activated T cells (NFAT) are a group of related proteins involved in distal TCR signaling. NFAT1, a member of the NFAT family, is required for T cell activation following TCR engagement. The mechanism of NFAT activation is complex and is mediated by multiple cellular factors which have been extensively reviewed [31, 32]. Briefly, NFAT proteins are phosphorylated by various cellular kinases in resting T cells and reside in the cytoplasm as an inactive transcription factor [31, 32]. Following TCR engagement, NFAT proteins are dephosphorylated by the calcium-dependent serine phosphatase calcineurin. Upon dephosphorylation, the NFAT proteins are activated and translocate to the nucleus as active transcription factors and induce NFAT-dependent gene expression required for T cell activation [31, 32].

Since NFAT proteins play a key role in distal TCR signaling, and previous studies suggest that SFKs may regulate distal TCR signaling, we characterized the role of active SFKs in resting T cells and identified a novel mechanism by which SFKs prevent aberrant NFAT1 activation in a calcineurin dependent manner.

## Materials and methods

### Cells

Jurkat T cell lines (clone E6.1) and the Lck-deficient (JCaM1.6) were obtained from ATCC. JCaM1.6 cells with Lck were kindly provided by Dr. Jon Houtman (University of Iowa) and HuT78 cells were kindly provided by Dr. Raj Puri (CBER/FDA). Jurkat cell lines were maintained in RPMI 1640 supplemented with 10% heat-inactivated fetal calf serum (hFBS), 2mM L-glutamine, 100 IU/ml penicillin, and 100 μg/ml streptomycin. HuT78 cells were cultured in IMDM media, supplemented with 20% hFBS, 2mM L-glutamine, 100 IU/ml penicillin, and 100 μg/ml streptomycin.

### Peripheral blood mononuclear cells (PBMCs) isolation

Whole blood was obtained from healthy donors at the National Institutes of Health (NIH) Blood Bank. All the donors provided written consent for their blood products to be used in research projects. All the samples provided to the investigators were de-identified. This study was exempted by the FDA’s IRB, Research Involving Human Subject Committee (RIHSC). PBMCs were isolated from whole blood using Ficoll-Hypaque gradient centrifugation. Isolated PBMCs were washed in cold PBS, and resuspended in complete RPMI and incubated overnight at 37°C. The following day, T cells in suspension were harvested from the culture media and the adherent cells were discarded. Up to 60% of cells in the final culture were CD3+ T cells.

### Inhibitors

Src-kinase inhibitor PP2 (Selleck Chemicals) and Lck-inhibitor II (EMD Millipore) were used. Inhibitors were suspended in DMSO and cells treated with DMSO alone served as controls. Unless otherwise noted, to achieve optimum inhibition of kinase activity, Jurkat T cells and primary PBMCs were treated with PP2 at concentrations of 5μg/ml and 1μg/ml respectively. Lck-inhibitor II was used at 10μg/ml. Calcineurin inhibitors FK506 and Cyclosporin A (CsA) were obtained from Selleck Chemicals and were used at 1μM concentration. Cells were treated with the inhibitors for 18 hours before stimulation.

### Cell stimulation

Jurkat T cells and primary human PBMCs (1×10^6^ cells/ml) were treated with the inhibitors or DMSO alone. Cells were counted using the Countess II FL automated cell counter (Invitrogen) and cell viability was determined using the trypan blue exclusion method. Following overnight incubation, cells were stimulated with phorbol 12-myristate 13-acetate (PMA, 50ng/ml) and ionomycin (1 μg/ml). Following 24 hours of stimulation, cellular receptor expression and cytokines were measured by flow cytometry and ELISA respectively. To assess proximal TCR activation, cells were treated with the inhibitor for 1 hour at 37°C, and stimulated with anti-CD3 (5μg/ml) or PMA/ionomycin for 2 minutes. Activation induced phosphorylation of Lck, ZAP-70 and LAT were assessed by immunoblot analysis.

### NFAT-luciferase assay

pGL3-NFAT firefly luciferase plasmid was a gift from Dr. Jerry Crabtree (Addgene plasmid # 17870) and plasmid encoding renilla luciferase was kindly provided by Dr. Jakob Reiser (CBER/FDA). Cells were co-transfected using the Amaxa Cell Line Nucleofector Kit V, program X-005, with pGL3-NFAT firefly luciferase encoding plasmid along with renilla luciferase as a control. Following transfection, cells were either treated with the inhibitor or the DMSO control. Luciferase activity was measured 48 hrs post transfection using Promega’s Dual-Glo Luciferase kit following manufacture’s protocol. Firefly luciferase activity was normalized to the renilla luciferase activity.

### Flow cytometry

Cellular receptor expression was measured with CD3 (V450), CD4 (A700), CD8 (FITC) and CD25 (PE) (BD Biosciences) using the manufacturer’s recommendation. Cells were incubated on ice for 1 hour and washed with PBS. Data was acquired on BD LSR II flow cytometer using single stained CompBeads (BD Biosciences) for compensation. Live cells were gated based on forward and side scatter. For propidium iodide (PI) staining, cells were incubated with PI for 10 minutes before analysis and at least 10,000 total events were collected in each experiment and the FlowJo program (Tree Star Inc.) was used for data analysis. All flow cytometry experiments were repeated at least three times with consistent results.

### ELISA

IL-2 and IFN-γ released into cell culture supernatant was quantified using cytokine specific human ELISA kit (BD Biosciences) according to the manufacturer’s instructions.

#### Subcellular fractionation

Fractionations were performed using 8-12x10^6^ cells per sample using a modified REAP detergent based method as previously described [33, 34]. Cells were gently washed in cold PBS and resuspended in buffer A: 10 mM KCl, 10 mM MgCl2, 10 mM HEPES, 1 mM EDTA, 1 mM DTT, EDTA-free complete protease/phosphatase inhibitors, 0.5% NP-40. Following 10 minutes incubation on ice; cells were spun at 5,000 g at 4°C for 5 minutes. Supernatant with enriched with cytoplasmic fractions were collected and the remaining cell pellet containing nuclear proteins was washed 1x in buffer A prior to resuspension in buffer B: 450 mM NaCl, 1.5 mM MgCl2, 20 mM HEPES, 0.5 mM EDTA, 1 mM DTT, EDTA-free, complete protease/phosphatase inhibitors. Following 10 minutes incubation on ice; the resuspended cell pellet was spun at 20,000 g (4°C for 10 minutes). The nuclear protein in the supernatant was collected.

### Immunoblot analysis

Cellular lysates were mixed with Laemmli sample buffer, heated at 95°C for 5 minutes and separated by polyacrylamide gel electrophoresis and transferred to nitrocellulose membranes using iBlot transfer system. Membranes were incubated in 10% fat-free dry milk for 1 hour at room temperature followed by incubation with primary antibodies. Proteins were detected with Super Signal West Dura (Thermo Scientific 34075) using a Bio-Rad ChemiDoc MP imaging system. Immunoblots were quantified using ImageJ (NIH). Primary antibodies used were: pLck (Y394, R&D Systems), pZAP-70 (Y319), total Lck and lamin A/C from Cell Signaling, pLAT(Y226), total ZAP-70, and NFAT-1 from BD Biosciences, total LAT from Biolegend, GAPDH from Invitrogen, beta-actin from Sigma and CD3 (OKT3) from Thermo Fisher.

### Statistics

Statistics were performed using GraphPad software V7.0 (GraphPad Software Inc.). Two-way ANOVA was used to compare results from multiple groups and two-sided Student’s t test was used to compare results from two groups and *P* values less than 0.05 were considered statistically significant.

## Results

### Src-family of kinases (SFKs) regulate distal T cell receptor (TCR) signaling in human T cell lines

**Fig 1:**
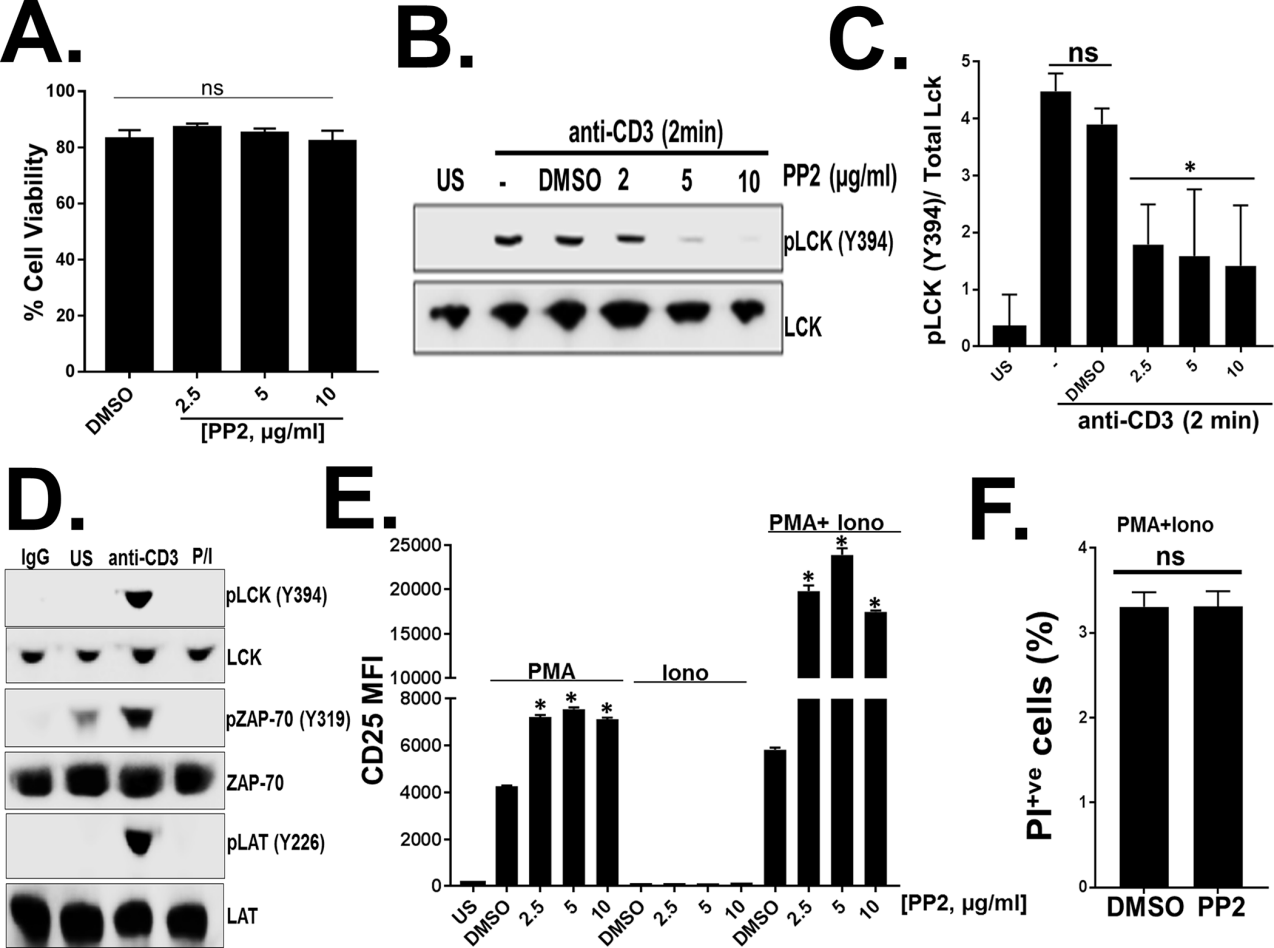
Inhibition of Src-family kinases (SFKs) by PP2 enhances distal T cell receptor (TCR) signaling. (A) Jurkat cell viability was measured following 18 hours treatment with various concentrations of PP2 by trypan blue exclusion method. (B) Activation of SFKs was measured in Jurkat T cells by assessing Lck phosphorylation at tyrosine 394 (Y394) following anti-CD3 stimulation (2 minutes) in presence of various concentrations of PP2, DMSO or culture media (-) for 30 minutes. (C) Quantification of immunoblots from panel B obtained from 3 independent experiments. (D) Immunoblot analysis of phosphorylated Lck (Y394), ZAP-70 (Y319) and LAT (Y226) following 2 minutes of stimulation with isotype control (IgG), anti-CD3 IgG or PMA/Ionomycin. (E) CD25 surface expression was measured in Jurkat T cells following 18 hours of DMSO alone or PP2 treatment and 24 hours of PMA, Ionomycin or PMA/Ionomycin stimulation by flow cytometry. (F) Propidium iodide staining of Jurkat T cells following 18 hours of DMSO or PP2 treatment and 24 hours of PMA/Ionomycin stimulation. US = Unstimulated. Data represent the average of three technical replicates, and the standard deviation is shown. Each experiment was independently performed three times with similar results. *P< 0.01, ns = not significant.

Inhibition of Src-family of kinases (SFKs) blunts the proximal TCR-mediated signaling pathways [20, 21]. Activation of T cells with phorbol 12-myristate 13-acetate (PMA) and ionomycin (P/I) activates the distal TCR signaling pathways mediated by the protein kinase C (PKC) and NFAT1, and bypasses the requirement of SFKs [4, 35]. To assess the role of active SFKs on distal TCR signaling, Jurkat T cells were incubated with SFKs-specific inhibitor PP2 or the DMSO control [36]. Although PP2 is a SFK-specific inhibitor, it has been shown to inhibit other cellular kinases at higher concentrations [37, 38]. PP2 treatment did not affect cell viability as measured by trypan blue exclusion assay (Fig 1A). As expected, PP2 treatment inhibited activation of the Src-kinase Lck in a dose-dependent manner within 30 minutes (Fig 1B and 1C). Vehicle (DMSO) treatment alone did not affect Lck phosphorylation (Fig 1B and 1C). Lck phosphorylation was not detected in cells treated with PP2 for longer than 1 hour, even at low (2.5 μg/ml) concentrations, suggesting complete Lck inhibition. Consistent with the previous observations [4, 35], P/I stimulation bypassed the proximal TCR signaling as only anti-CD3 but not P/I stimulation activated Lck (Y394), ZAP-70 (Y319) and LAT (Y226) (Fig 1D). Following stimulation of distal TCR-mediated signaling with P/I, T cell activation was measured by assessing CD25 surface expression. CD25 expression was low in unstimulated (US) cells (Fig 1E).PP2 treatment and P/I stimulation enhanced CD25 surface expression in a dose-dependent manner compared to the cells treated with DMSO control (Fig 1E). Stimulation with PMA alone also resulted in an increase in CD25 surface expression in PP2-treated cells compared to DMSO treated cells; however, ionomycin alone did not induce CD25 expression (Fig 1E). PP2-treatment and P/I stimulation did not affect cell viability as measured by propidium iodide (PI) staining (Fig 1F).

**Fig 2:**
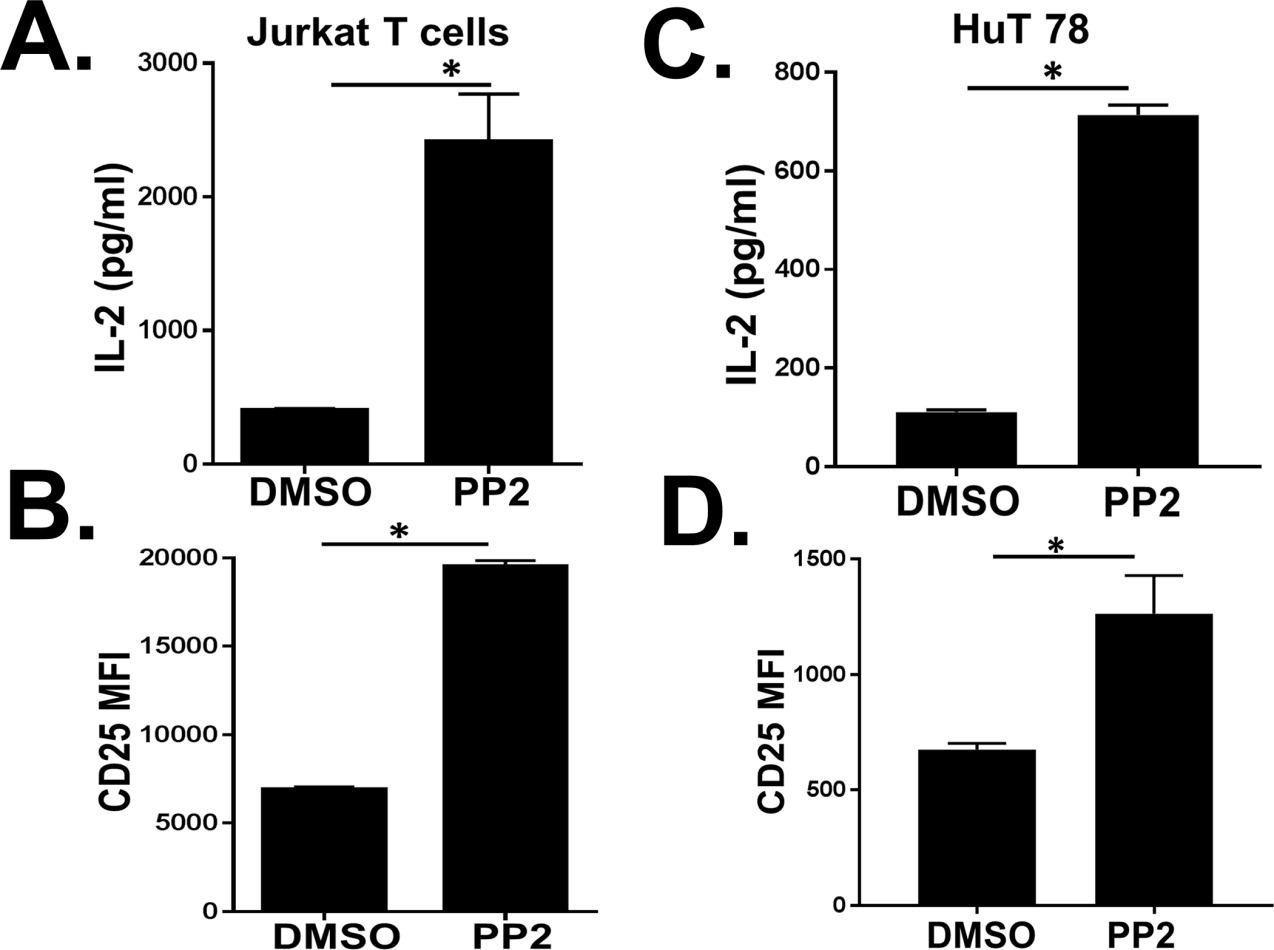
Inhibition of Src-family kinases (SFKs) enhances distal T cell receptor (TCR) signaling in two different human T cell lines. Activation of distal TCR signaling was measured in two different human T cell lines by assessing (A, C) IL-2 release and (B, D) CD25 surface expression following DMSO or PP2 (5μg/ml) treatment and PMA/Ionomycin stimulation. Jurkat cells = panels A and B. HuT78 = panels C and D. Data represent the average of three technical replicates, and the standard deviation is shown. Each experiment was independently performed three times with similar results. *P< 0.01.

To further assess the effect of SFKs inhibition on human T cells, two different human T cell lines (Jurkat and HuT78) were treated with the Src-inhibitor PP2 or the DMSO control. Following P/I stimulation, T cell activation was measured by assessing IL-2 release and CD25 expression. SFKs inhibition significantly enhanced IL-2 release and CD25 expression in both Jurkat (Fig 2A and 2B) and HuT78 T cells (Fig 2C and 2D) compared to the cells treated with DMSO alone.

Together, these data demonstrate that inhibition of SFKs enhances distal TCR-mediated signaling pathways in human T cells stimulated by P/I.

### Src-family of kinases (SFKs) regulate NFAT1 signaling in unstimulated Jurkat T cells

As noted, stimulation of distal TCR-mediated signaling by P/I bypasses the proximal TCR-signaling (Fig 1C) and selectively activates the nuclear factor of activated T cells (NFAT) and the protein kinase C (PKC) pathways [4, 35]. NFAT proteins are essential transcription factors required for the activation of T cells, and inhibition of NFAT1 inhibits T cell activation and effector function [32]. In resting T cells, NFAT proteins are phosphorylated and reside in the cytoplasm as inactive transcription factors. Following stimulation, NFAT proteins are dephosphorylated by calcineurin and translocate to the nucleus where they activate gene expression required for T cell function [32].

**Fig 3:**
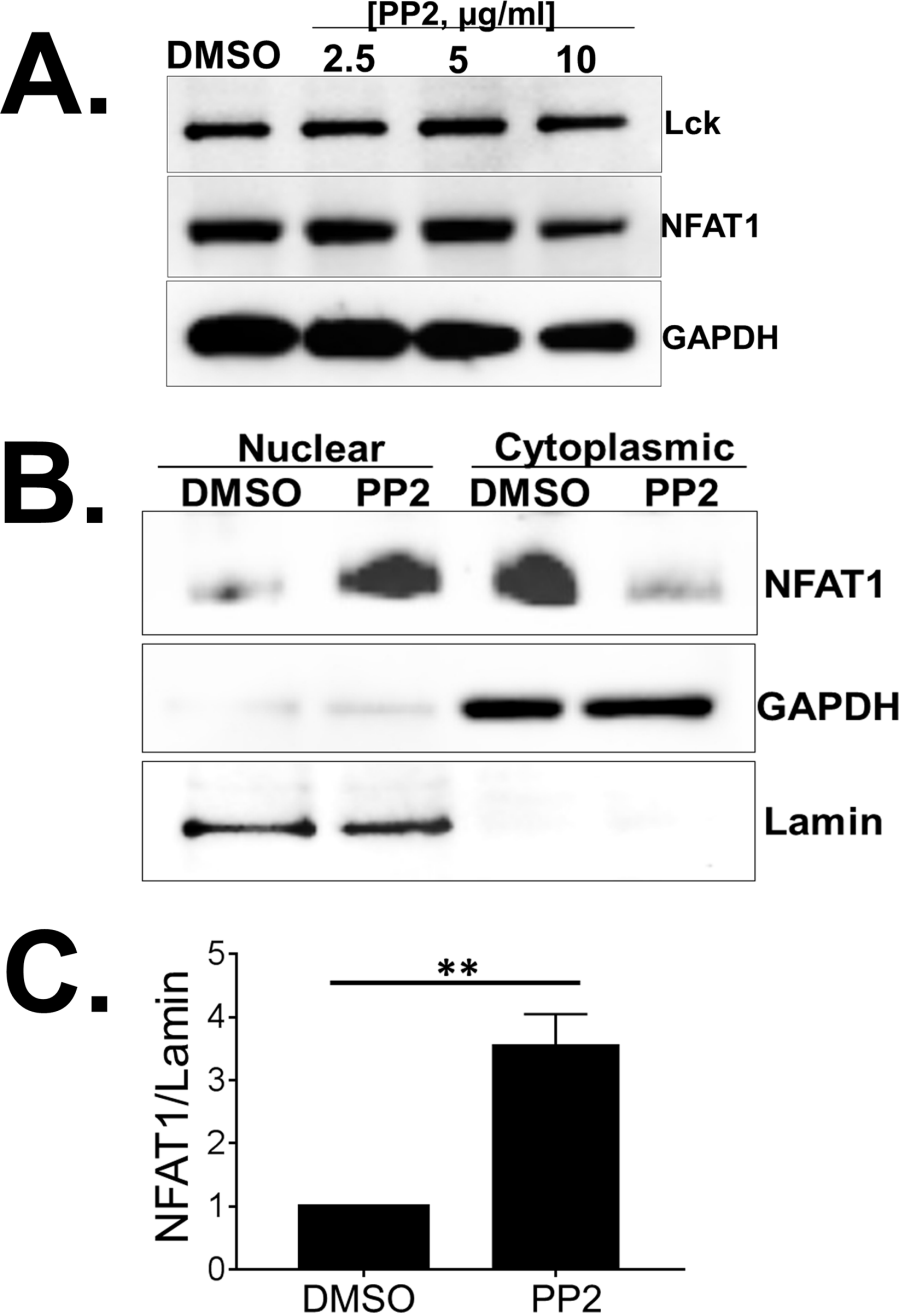
Src-family kinases (SFKs) regulate NFAT1 signaling in unstimulated Jurkat T cells. (A) Immunoblot analysis of Src-kinase Lck and NFAT1 protein expression in Jurkat T cells treated with various concentrations of PP2. (B) Immunoblot analysis of subcellular localization of NFAT1, GAPDH and Lamin in Jurkat T cells treated with DMSO or PP2. GAPDH and Lamin served as a loading control for cytoplasmic and nuclear fractions respectively. (C) Quantification of NFAT1 protein in the nuclear fraction of Jurkat T cells either treated with DMSO or PP2. Amount of NFAT1 protein in the nuclear fraction was normalized by the Lamin protein level. US = unstimulated, P+I = PMA/ Ionomycin. Data represent the average of three technical replicates, and the standard deviation is shown. Each experiment was independently performed three times with similar results. *P< 0.05; **P< 0.01.

Since PP2-mediated inhibition of SFKs enhanced P/I mediated distal TCR signaling, we assessed the effect of SFK inhibition on NFAT1 expression. PP2 treatment did not alter the expression of NFAT1 or the Src-kinase Lck in Jurkat T cells (Fig 3A). Next, we assessed the effect of SFK inhibition on subcellular localization of NFAT1. Jurkat T cells treated with either DMSO or PP2 were subjected to subcellular fractionation. Cytoplasmic and nuclear proteins were isolated and analyzed by immunoblotting. The purity of subcellular fractions was assessed by immunoblotting for GAPDH (cytoplasmic) and Lamin (nuclear). Highly pure cytoplasmic and nuclear fractions were obtained (Fig 3B). Inhibition of SFKs in resting T cells significantly increased NFAT1 protein in the nuclear fraction with a concomitant reduction in the cytoplasmic fraction (Fig 3B and 3C). As expected, in DMSO treated cells the majority of NFAT1 was detected in the cytoplasmic fraction (Fig 3B).

Together, these data demonstrate that inhibition of SFKs aberrantly activates NFAT1 in unstimulated Jurkat T cells resulting in nuclear translocation of a pool of NFAT1 and resultant increase in NFAT1-dependent target gene expression.

### Lck prevents aberrant NFAT1 activation in unstimulated Jurkat T cells

**Fig 4:**
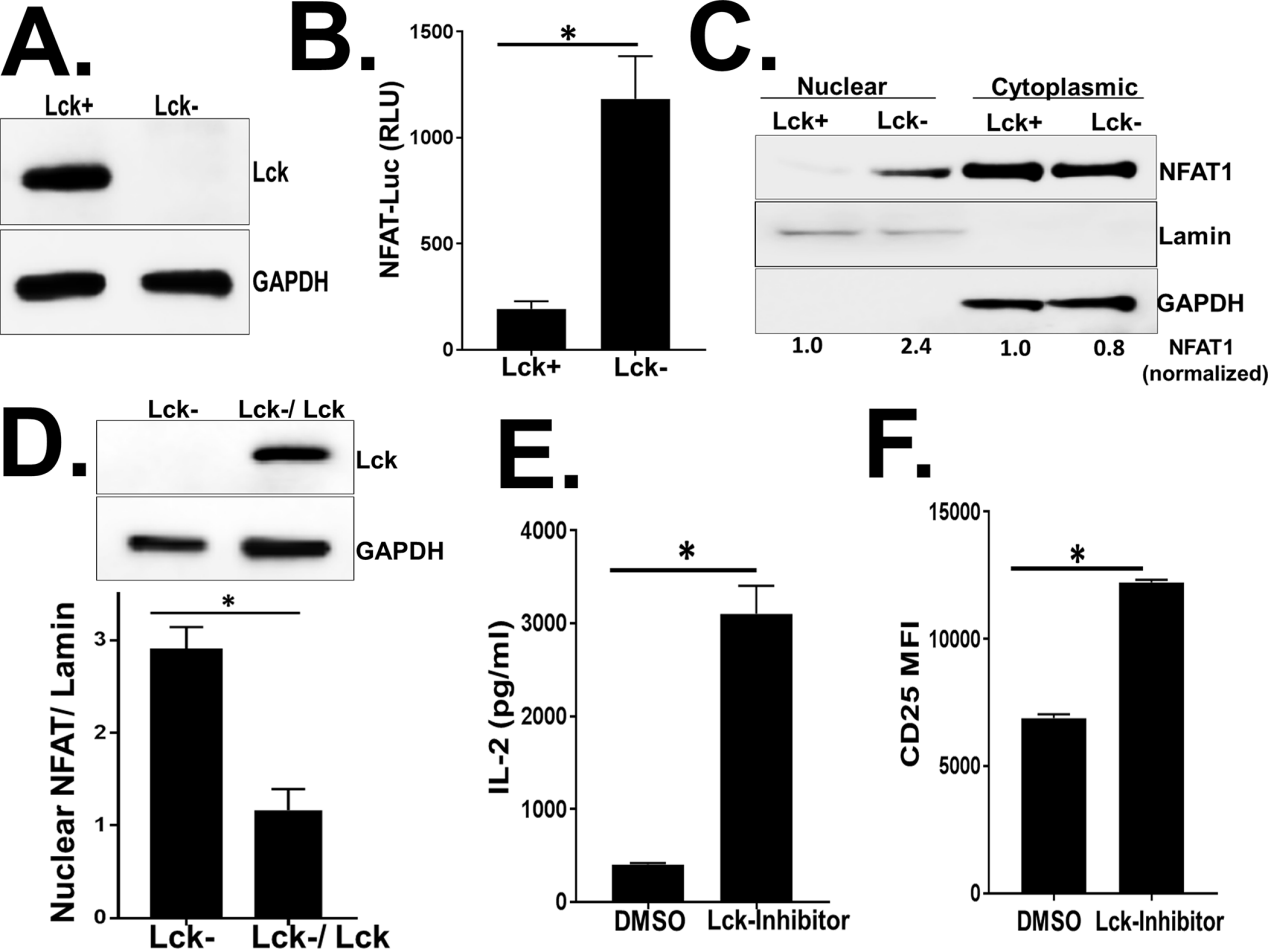
Src-kinase Lck prevents aberrant NFAT1 activation in unstimulated Jurkat T cells. (A) Immunoblot analysis of Lck expression in Jurkat T cells either expressing (Lck+) or not expressing (Lck-) Src-kinase Lck. GAPDH served as a loading control. (B) NFAT1-dependent firefly luciferase activity (NFAT1-Luc) was measured in Lck+ or Lck- Jurkat T cells. Firefly luciferase activity was normalized to renilla luciferase activity. (C) Immunoblot analysis of subcellular localization of NFAT1, GAPDH and Lamin in Lck+ or Lck- Jurkat T cells. GAPDH and Lamin served as a loading control for cytoplasmic and nuclear fractions respectively. NFAT1 protein levels in the cytoplasmic fraction and nuclear fractions were normalized with GAPDH and Lamin protein levels respectively. (D) Immunoblot analysis of NFAT in nuclear fraction obtained from Lck-deficient (Lck-) and Lck expression rescued (Lck-/Lck) Jurkat T cells. Activation of distal TCR signaling was measured by assessing (E) IL-2 release and (F) CD25 surface expression in Lck+ Jurkat T cells following treatment with DMSO or Lck-specific inhibitor (Lck inhibitor II) and PMA/Ionomycin stimulation. RLU = relative luciferase units. Data represent the average of three technical replicates, and the standard deviation is shown. Each experiment was independently performed three times with similar results. *P< 0.01.

Lck and Fyn are the major SFKs present in human T cells, and activation of Lck and Fyn is required for the proximal TCR-mediated signaling events [21]. PP2-treatment inhibits the enzymatic activity of both Lck and Fyn in human T cells [36]. To further characterize the SFKs involved in preventing aberrant NFAT1 activation, we studied Jurkat T cells either expressing Lck (Lck+ve) or lacking Lck expression (Lck-ve, JCam1.6) (Fig 4A). First, we assessed the NFAT1-dependent IL-2 promoter activity in unstimulated resting Lck+ve and Lck-ve Jurkat T cells as described above. Consistent with the previous observation that inhibition of SFKs enhanced NFAT1-dependent target gene expression (Fig 2), Jurkat T cells lacking Lck (Lck-ve) had significantly increased NFAT1-dependent IL-2 promoter activity as measured by the luciferase gene expression compared to the Lck+ve Jurkat T cells (Fig 4B).

Next, unstimulated resting Lck+ve and Lck-ve cells were subjected to subcellular fractionation and cytoplasmic and nuclear proteins were isolated and analyzed by immunoblot analysis. In unstimulated Lck+ve Jurkat T cells, NFAT1 was predominantly detected in the cytoplasmic fraction (Fig 4C). However, in Jurkat T cells lacking Lck expression (Lck-ve), NFAT1 was significantly enriched in the nuclear fraction (Fig 4C). The increased level of NFAT1 in the nuclear fraction observed in Lck-deficient Jurkat cells (Lck-) was significantly reduced by expressing Lck in those cells (Fig 4D).

To further assess the role of Lck in regulating distal TCR signaling, Lck+ve Jurkat T cells were treated with the Lck-specific inhibitor (Lck inhibitor-II) [39]. Following P/I stimulation, IL-2 release and CD25 surface expression were significantly enhanced in Jurkat T cells treated with the Lck inhibitor compared to the DMSO treated cells (Fig 4D and 4E). IL-2 release was not different in vehicle (DMSO) or untreated cells suggesting that DMSO was not responsible for the altered NFAT nuclear translocation (data not shown). This is consistent with the results obtained with Jurkat T cells treated with PP2 (Fig 3B) and suggest that the loss of Lck expression or loss of enzymatic function results in aberrant NFAT1 activation and nuclear translocation. Together, these data identify a novel role of Lck in negatively regulating distal TCR signaling in resting human T cells.

### Active Src-family kinases (SFKs) prevent aberrant NFAT1 activation in resting primary human T cells

**Fig 5:**
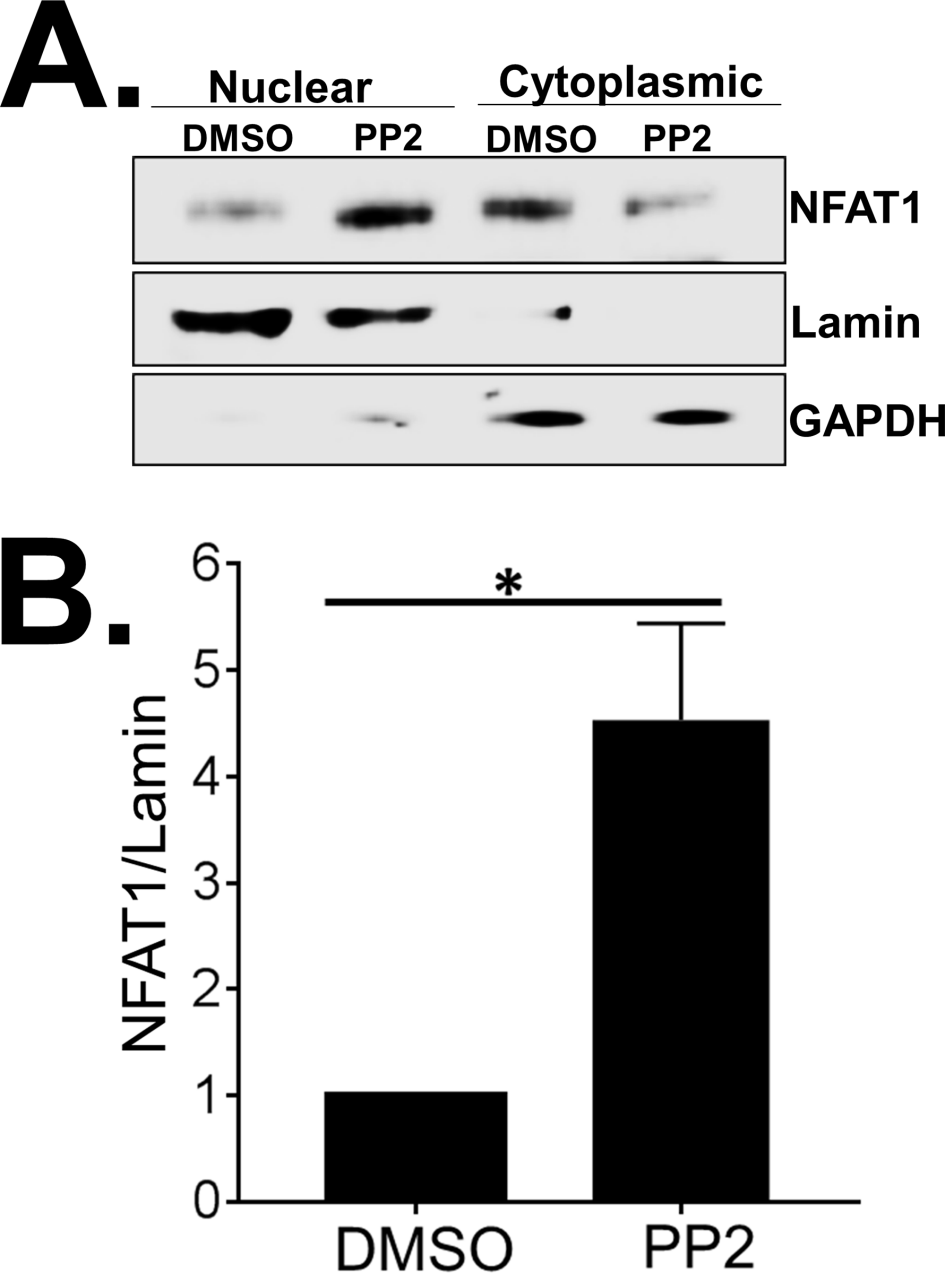
Active Src-family kinases (SFKs) prevent aberrant NFAT1 nuclear translocation in resting primary human T cells. (A) Immunoblot analysis of subcellular localization of NFAT1, GAPDH and Lamin in primary human T cells treated with DMSO or PP2. GAPDH and Lamin served as a loading control for cytoplasmic and nuclear fractions respectively. (B) Quantification of the NFAT1 protein in the nuclear fraction of primary human T cells obtained from four healthy blood donors either treated with DMSO or PP2. Amount of NFAT1 protein in the nuclear fraction was normalized by the Lamin protein. Each experiment was independently performed with four different donors with consistent results. *P < 0.01.

Recent studies identified a pool of active SFKs in resting T cells and suggest this pool plays an important role in proximal TCR signaling [2, 14, 24]. To assess the role of active SFKs in regulating distal TCR signaling in unstimulated T cells, we characterized the effect of SFK inhibition on NFAT1 activation in primary human T cells. Primary human T cells obtained from four different healthy blood donors were treated with PP2 or the DMSO control. Similar to Jurkat T cells (Fig 3B and 3C); inhibition of the active pool of SFKs in resting primary human T cells resulted into a significant increase in the nuclear translocation of NFAT1 (Fig 5A and 5B).

**Fig 6:**
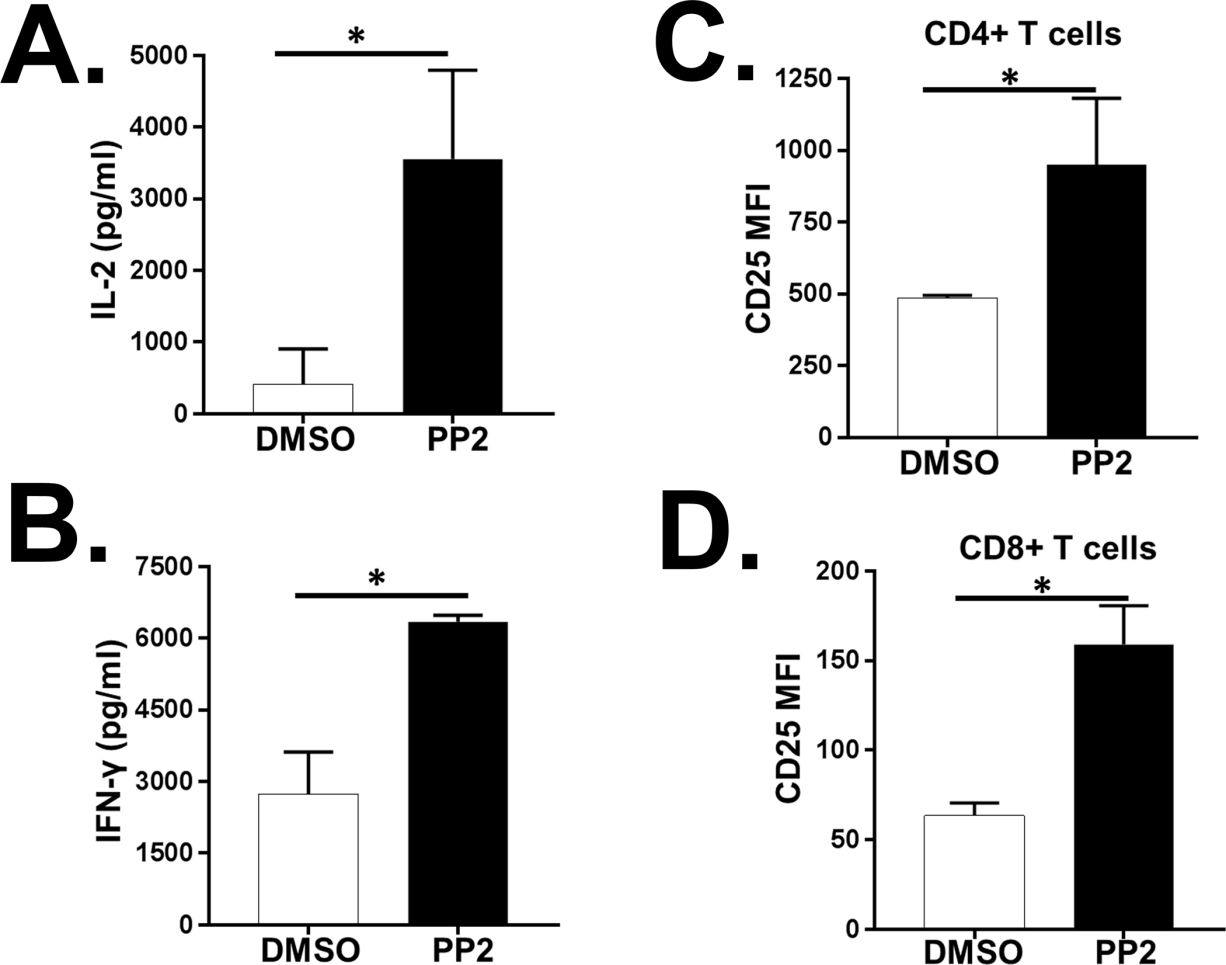
Inhibition of Src-family kinases (SFKs) enhances distal T cell receptor (TCR) signaling in primary human T cells. Primary human T cells obtained from healthy blood donors were treated with DMSO alone or PP2. Following stimulation with PMA/ Ionomycin, distal TCR signaling was measured by assessing IL-2 (A), IFN-γ (B) release and CD25 surface expression on CD4+ (C) and CD8+ (D) T cells. MFI = mean fluorescent intensity. Data represent the average of three technical replicates, and the standard deviation is shown. Each experiment was independently performed with three different donors with similar results. *P< 0.01.

To assess the effect of nuclear NFAT1 in primary T cells treated with PP2, T cell activation was measured following stimulation of distal TCR signaling with P/I. T cell activation was significantly enhanced in primary human T cells treated with PP2 compared to the DMSO control as measured by IL-2 and IFN-γ release (Fig 6A and 6B) and CD25 surface expression (Fig 6C and 6D). Consistent with the previous observation (Fig 1A), DMSO and PP2 treatment did not affect cell viability (data not shown).

Together, these data suggest that inhibition of the active pool of SFKs in resting primary human T cells enhances NFAT1 nuclear translocation and NFAT1-dependent target gene expression.

### Calcineurin inhibitors rescue aberrant NFAT1 activation in resting primary human T cells lacking active SFKs

**Fig 7:**
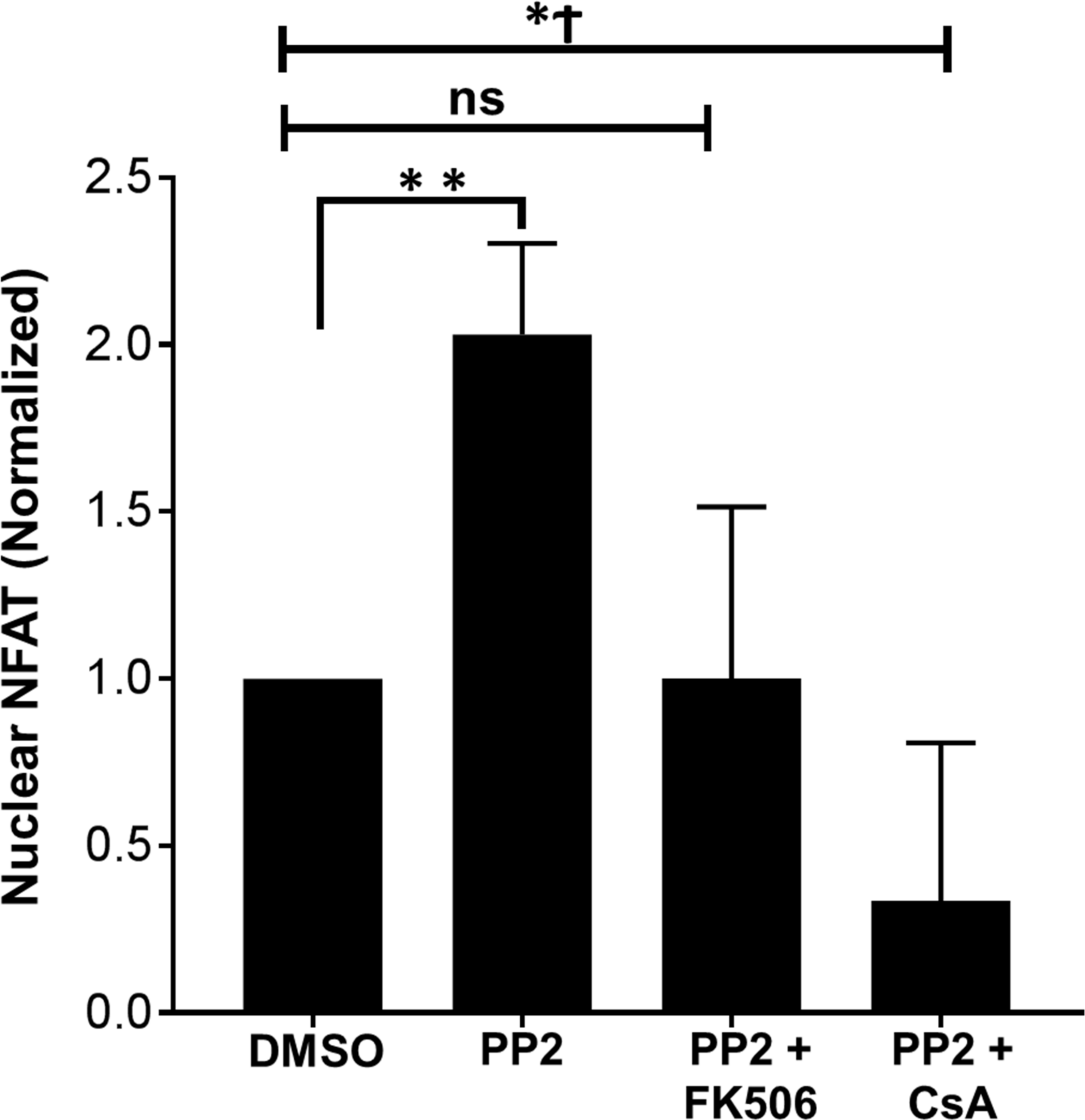
Calcineurin inhibitors prevent aberrant NFAT1 nuclear translocation in resting primary human T cells lacking active SFKs. Primary human T cells obtained from healthy blood donors were treated with DMSO alone or PP2 along with FK506 or Cyclosporin A (CsA). Following subcellular fractionations, NFAT1 protein in the nuclear fraction was quantified and normalized to the nuclear actin levels. Data represent the average obtained from three different donors along with the standard deviation. *P< 0.05, **P< 0.01, Ϯ = average obtained from two donors.

To understand the mechanism of altered NFAT1 function and nuclear translocation in resting human T cells lacking a pool of active SFKs, we assessed the role of calcineurin, the phosphatase that activates NFAT1 in human T cells [40]. Unstimulated primary human T cells were treated with two different calcineurin-specific inhibitors (FK506 and Cyclosporin A [CsA]) along with the SFK inhibitor PP2. Cells were subjected to subcellular fractionation and nuclear proteins were isolated. Consistent with previous observations (Figs 3B, 4C and 5A), inhibition of SFKs by PP2 resulted in an increase in nuclear NFAT1 (Fig 7). This increase in nuclear NFAT1 was not observed in T cells treated with PP2 in combination with calcineurin inhibitors FK506 and CsA. Thus, these data suggest that loss of active SKFs in resting T cells induce NFAT1 activation in a calcineurin-dependent manner.

## Discussion

T cell receptor (TCR) activation is the first essential step required to generate an effector T cell response [13, 41]. Src-family of tyrosine kinases (SFKs) are required for signaling through the TCR and T cell activation [21, 42], and inhibition of SFKs function impair proximal signaling initiated by the TCR [13, 14, 21]. Although, TCR signaling is required for T cell activation, proliferation and effector functions, persistent TCR signaling reduces T cell function [5, 6, 43–45]. Thus, TCR signaling is tightly regulated by various mechanisms to maintain T cell homeostasis [7–11, 46–50].

In resting T cells, the majority of SFKs are enzymatically inactive; however, recent studies have identified a pool of active SFKs (Lck and Fyn) in resting T cells [14, 24, 25] Although the significance of this pool of active SFKs remains unclear, they appear to contribute to proximal TCR signaling [14]. Phosphorylation of the carboxy-terminal tyrosine residue of the majority of SFKs by Csk inhibits its kinase activity and maintains T cells in a quiescent state [26]. This negative regulation of the proximal TCR signaling in resting T cells requires phosphorylation of the Csk-binding protein (Cbp) by an active pool of SFKs, and interactions between phosphorylated Cbp and Csk [26]. However, Cbp-deficient mice did not show any developmental defect and the T cell response in these mice were normal [27, 28]. This suggests that either Cbp is dispensable or other cellular factors compensate for loss of Cbp in T cells.

**Fig 8:**
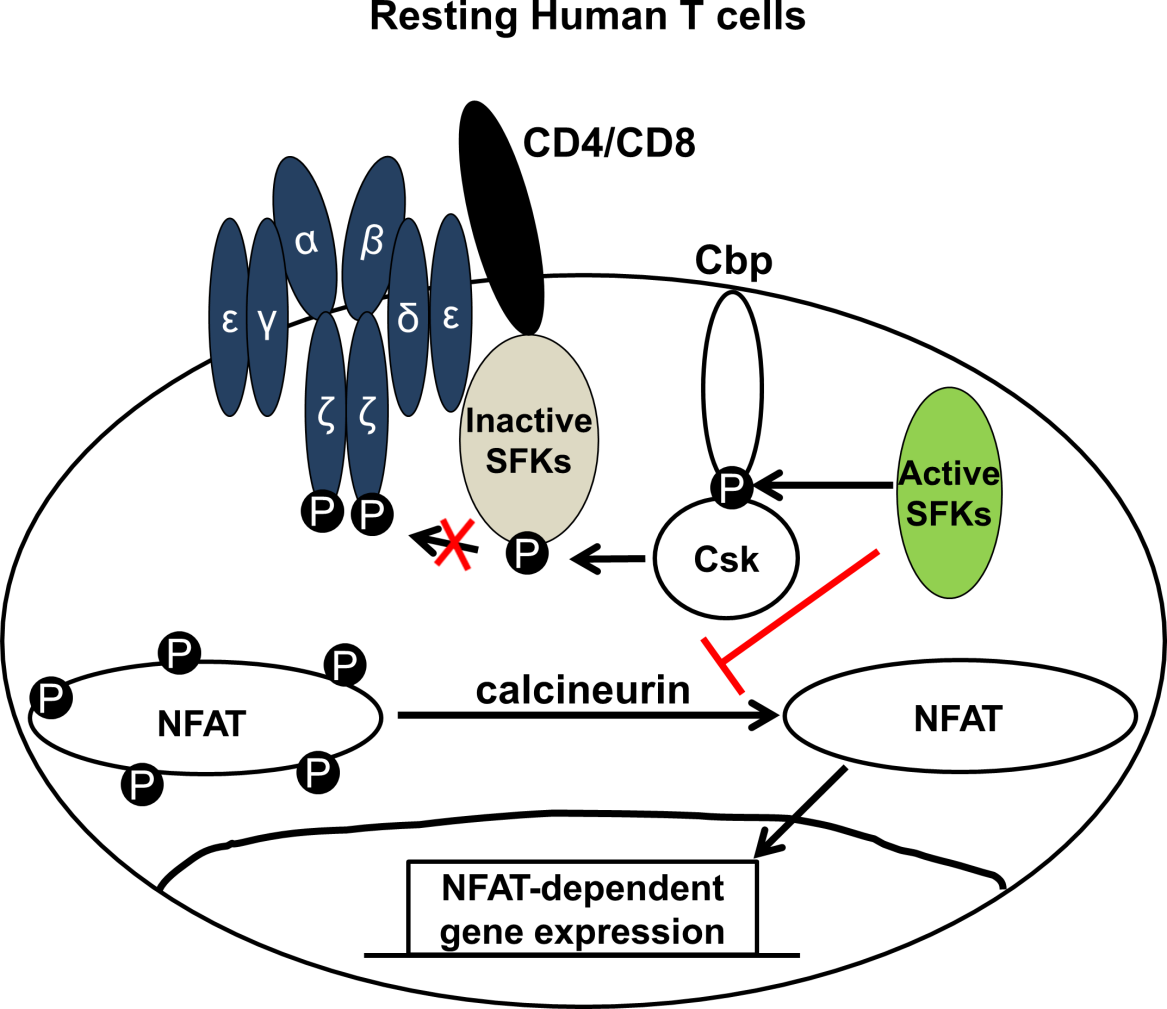
Proposed model for the role of active SFKs in resting human T cells. In resting human T cells, a pool of active Src-family kinases (SFKs) phosphorylates the Csk-binding protein (Cbp). Csk bound to the phosphorylated Cbp phosphorylates the majority of SFKs at the inhibitory C-terminal tyrosine. SFKs phosphorylated at the C-terminal inhibitory tyrosine are enzymatically inactive and cannot mediate proximal T cell receptor (TCR) signaling in absence of TCR stimulation. In addition, this active pool of SFKs also prevents aberrant NFAT1 activation and negatively regulates NFAT1-mediated distal TCR signaling in resting human T cells by suppressing calcineurin activity. Together, negative regulation of both proximal and distal TCR signaling by active SFKs contribute to maintain T cells in a quiescent state in absence of TCR stimulation.

In this study, we found that the active pool of SFKs negatively regulated distal TCR signaling (Fig 8). Distal TCR signaling was enhanced in T cell lines and primary human T cells treated with SFK-specific inhibitors. The enhanced distal TCR signaling was due to nuclear translocation of the transcription factor NFAT1. NFAT1 is phosphorylated by many cellular kinases, and phosphorylated NFAT1 remains in the cytoplasm in an inactive form [32]. Upon TCR activation, NFAT1 is dephosphorylated by the phosphatase calcineurin, leading to a conformational change and exposure of a nuclear localization signal (NLS) that facilitates nuclear translocation [32]. When T cells were treated with PP2, NFAT1 nuclear translocation increased by approximately 4-fold compared to the DMSO treated cells, suggesting that SFKs negatively regulate NFAT1 function in resting T cells. In a human T cell line lacking Src-kinase Lck expression, NFAT1 nuclear localization and target gene expression increased by about 2.4-fold. We interpret this to suggest that Lck negatively regulates NFAT1 in resting T cells; however, PP2 may have effects on other cellular kinases, and other cellular factors may be involved in NFAT1 regulation in resting T cells.

The increased nuclear localization of NFAT in Lck negative cells suggest that regulation of distal TCR signaling is mediated in part by Lck. Loss of other Src-kinase (Fyn) results in impaired proximal TCR signaling in resting T cells [26]. Thus, Fyn and Lck appear to regulate both proximal and distal TCR signaling in resting human T cells. The altered localization of NFAT1 in resting T cells treated with PP2 was corrected by the calcineurin inhibitors Cyclosporin A and FK506 suggesting active SFKs may negatively regulate calcineurin activity and prevent aberrant NFAT1 activation.

Negative regulation of both proximal and distal TCR signaling in resting T cells is important in maintaining T cell homeostasis. Src-kinases Lck and Fyn have been shown to be essential for survival of naïve T cells, and TCR signaling is important for homeostasis of CD4 memory cells [51, 52]. An increase in Fyn activity impairs TCR signaling and correlates with T cell anergy [26, 52–54]. Here, we found that inhibition of SFK activity or the loss of Lck expression resulted into increased NFAT1 activation in unstimulated Jurkat T cells and resting primary T cells. Thus, a balance in Src-kinase activities appears to be critical for keeping T cells in a resting state in the absence of TCR ligands.

The mechanism for increased NFAT-1 nuclear translocation in the absence of active Src-kinases appears to be dependent upon calcineurin. Two different calcineurin inhibitors were able to restore NFAT-1 nuclear translocation in PP2-treated cells (Fig 7). These data suggest that the active pool of Src-kinases negatively regulate NFAT-1 via calcineurin. Consistent with this, a recent study found that calcineurin is recruited to the TCR complex, and that loss of calcineurin activity impaired Lck activation [55]. The effect of PP2 on NFAT-1 nuclear translocation may also be due to its effect on other cellular kinases. Thus, it is also possible that multiple factors contribute to the negative regulation of NFAT in resting T cells.

Since T cell activation is required to generate an effective adaptive immune response, and inappropriate activation plays a role in immunological disorders including autoimmune diseases, understanding Src-kinase mediated positive and negative regulation of TCR signaling may provide novel insights into human T cell signaling pathways, and aid in development of novel immunomodulatory therapeutics including effective T cell based therapies. Chimeric antigen receptor (CAR) T cells have shown a great potential to treat various human cancer. To maintain their therapeutic effect, CAR T cells must be activated upon binding to the target antigen. Identification of a novel role of active Src-kinases in negatively regulating NFAT activation and TCR signaling in the absence of a target antigen might aid in designing T cell therapies that does not perturb this pool and maintain T cell homeostasis during manufacturing of CAR-T cells.
